# Cancer Incidence Profiles in the Miyagi Cohort Study

**DOI:** 10.2188/jea.14.S7

**Published:** 2005-03-18

**Authors:** Yoshikazu Nishino, Yoshinori Suzuki, Kaori Ohmori, Atsushi Hozawa, Keiko Ogawa, Shinichi Kuriyama, Yoshitaka Tsubono, Daisuke Shibuya, Ichiro Tsuji, Akira Fukao, Shigeru Hisamichi

**Affiliations:** 1 Division of Epidemiology, Department of Public Health and Forensic Medicine, Tohoku University Graduate School of Medicine.; 2 Miyagi Cancer Society.; 3 Department of Public Health, Yamagata University School of Medicine.

**Keywords:** prospective cohort study, follow-up, cancer, incidence

## Abstract

BACKGROUND: There were few prospective cohort studies in Japan using cancer incidence as an endpoint.

METHODS: We conducted a baseline survey with two self-administered questionnaires regarding lifestyle and personality on the residents aged 40 to 64 years in 14 municipalities of Miyagi Prefecture, Japan, during June through August, 1990. Out of the eligible 51,921 residents, 47,605 (91.7%) responded to the lifestyle questionnaire and formed the cohort under study. We collated the list of subjects in the cohort with the Miyagi Prefectural Cancer Registry data through December 31, 1997. To identify the same person between two data, we used four personal characteristics (sex, name, birthday, and municipality of dwelling).

RESULTS: We ascertained 1,718 cases of incident cancer. In men, gastric cancer was the leading site of cancer (27.7%), followed by lung cancer and colon cancer. In women, breast cancer was the most common (19.6%), followed by gastric cancer and colon cancer.

CONCLUSIONS: By record linkage with regional cancer registry data, it becomes possible for us to investigate the effect of various life-styles on cancer incidence in the Miyagi Cohort Study. We expect this data to contribute to the progress of research on cancer etiology and cancer prevention.

The Miyagi Cohort Study is a population-based prospective cohort study of lifestyle variables and mortality and cancer incidence.^1^ The study cohort consists of 47,605 subjects aged 40-64 years living in 14 municipalities in Miyagi prefecture in rural northern Japan. We have been following up vital status and migration of the study cohort by the Residential Registration Record of each municipality and death certificate. When evaluating the etiology of cancer in a cohort study, mortality is frequently utilized as an endpoint instead of incidence. In this case, nonfatal cases are not taken into account and the results would be distorted. However, there have been few prospective cohort studies in Japan using cancer incidence as an endpoint. We have been following up cancer incidence by record linkage with the Miyagi Prefectural Cancer Registry, a regional cancer registry covering the study area. We describe here the method of follow-up for cancer incidence and profiles of it in this cohort. Also, in order to confirm whether epidemiologic study using cancer incidence as an endpoint in this cohort is proper, we investigated the relationship between smoking and lung cancer incidence which other cohort studies showed to be strong consistently^2^^-^^4^.

## METHODS

### Miyagi Prefectural Cancer Registry

The Miyagi Prefectural Cancer Registry has covered the entire prefecture from the start in 1959. Cancer cases are registered from clinics and hospitals (inpatients and outpatients), radiology and pathology departments, autopsy records, mass screening records and death certificates. About 40% of cases are reported from hospitals and clinics, and 60% of cases are collected by the registry personnel. Multiple primary cancers for the same persons are registered according to the IARC/IACR criteria^5^ with exception that colon cancers with different four digit codes of the International Classification of Diseases, 9th Revision (ICD-9)^6^ are regarded as multiple primaries and registered separately. The site and histology of each cancer are coded according to the International Classification of Diseases for Oncology, Second Edition (ICD-O2)^7^.

As an index of completeness of reporting in cancer registry, the use of proportion of cases first notified to the registry via the death certificate (DCN%) is proposed.^8^ In the Miyagi Prefectural Cancer Registry, DCN% from 1993 to 1997 was equal to DCO%, proportion of cases with death certificate only, in this period, which was 16% in men and 18% in women.^9^

### Method of Record Linkage

We collated the list of cohort members with the Miyagi Prefectural Cancer Registry data through December 31, 1997 to ascertain the cancer incidence of the subjects. Four characteristics were used to identify the cohort members in cancer registry data: name in Chinese characters (Kanji), sex, birthday, and municipality of dwelling. Regarding name, first name and last name each was compared between the list and registry data, and we collated birth year, birth month, birth date about birthday separately. In both lists, birth year was expressed in gengo, which is the method of expressing year usually used in Japan and is composed of two parts, name of an era (Meiji, Taisho, Showa, Heisei), and the year since the era started. We checked these eight variables (first name, last name, sex, name of era at the birth, year since the era started at the birth, birth month, birth date, and municipality of dwelling) to identify the same persons between the two lists. The check was firstly conducted by use of computer, which put out the list of persons whose data items were agreement about the six variables and over as candidates for identical persons. We further checked the list manually and judged whether these candidates were the identical persons or not.

### Smoking and Lung Cancer Incidence

We investigated the relationship between smoking and the incidence of lung cancer among male cohort members. Because there were few cases for past and current smokers in women, we did not conduct analyses for women. With regard to the evaluation of smoking in the cohort members, we used the data of a baseline survey conducted by a self-administered questionnaire in 1990. The questionnaire asked firstly whether subjects were current, past, or never smokers. Current or past smokers were further asked about age of smoking initiation and the number of cigarettes smoked per day at baseline (current smokers) or in the past (past smokers). For past smokers, the time of smoking cessation was also asked. The method of a baseline survey was described elsewhere in detail.^1^

Person-years of follow-up for each subject were counted from June 1, 1990, until the date of diagnosis of lung cancer, the date of emigration outside study areas, the date of death, or the end of the study period (December 31, 1997), whichever occurred first. We computed relative risk (RR) of lung cancer incidence according to categories of smoking status, the number of cigarettes smoked per day and pack-years of smoking, using Cox proportional-hazards regression, employing the PHREG procedure on SAS^®^ version 8.2 statistical software package (SAS Institute Inc., Cary, NC, USA). We calculated age-adjusted RR and RR further adjusted for the following potentially confounding variables: education (up to 15 years of age, 16-18, 19 years or older); alcohol drinking (never drinkers, past drinkers, or current drinkers who consumed less than 22.8g, 22.8-45.5g, or 45.6 g or more alcohol per day); walking time per day (less than 1 hour, or 1 hour or longer); consumption frequencies of green vegetables and oranges (almost daily, 3-4 times per week, 1-2 times per week, or 1-2 times per month or less often). P values for tests of linear trend were estimated using the number of cigarettes per day or pack-years as a continuous variable. All P values were two-tailed.

## RESULTS

### Profile of Cancer Incidence

We ascertained 2,591 cases of cancer in 2,457 subjects excluding carcinoma in situ (CIS) among the 47,605 cohort members (22,836 men and 24,769 women) by collation with the Miyagi Prefectural Cancer Registry data. Of them, 2,332 had one, 118 had two, five had three and two subjects had four primary cancers. With regard to the date of diagnosis, 873 cases were diagnosed before June 1, 1990, when the follow-up started. Table 1 shows the number of cases for site-specific cancer by five-year age category at entry in 1,718 cases of cancer (1,023 in men and 695 in women) diagnosed from the start of follow-up (June 1, 1990) through December 31, 1997. In men, gastric cancer was the leading site of cancer (27.7%), followed by lung cancer (13.8%), colon cancer (12.6%), rectal cancer (7.7%) and esophageal cancer (6.3%). In all five-year age categories, gastric cancer was the most frequent cancer, and the proportion of lung cancer increased as age category became older. In women, breast cancer was the most common cancer (19.6%), followed by gastric cancer (17.7%), colon cancer (10.5%), lung cancer (7.8%) and thyroid cancer (7.5%). Breast cancer comprised over 40 % of total cancer in the age of 40-44, but the proportion decreased as age category became older.

**Table 1.** The number of cases and incidence^†^ for site-specific cancer by five year age category at entry.

	ICD-O2		Age at entry (year)											

			40-44		45-49		50-54		55-59		60-64		Total	
Men														
Person-years			41,712		27,198		28,875		33,842		35,178		166,804	

All sites	C00-C80	n	58	(100)	88	(100)	148	(100)	286	(100)	443	(100)	1,023	(100)
		Incidence		139.0		323.6		512.6		845.1		1259.3		613.3
Esophagus	C15	n	1	(1.7)	3	(3.4)	10	(6.8)	18	(6.3)	32	(7.2)	64	(6.3)
		Incidence		2.4		11.0		34.6		53.2		91.0		38.4
Stomach	C16	n	16	(27.6)	27	(30.7)	46	(31.1)	72	(25.2)	122	(27.5)	283	(27.7)
		Incidence		38.4		99.3		159.3		212.8		346.8		169.7
Colon	C18	n	9	(15.5)	11	(12.5)	16	(10.8)	37	(12.9)	56	(12.6)	129	(12.6)
		Incidence		21.6		40.4		55.4		109.3		159.2		77.3
Rectum	C19-C21	n	6	(10.3)	8	(9.1)	12	(8.1)	22	(7.7)	31	(7.0)	79	(7.7)
		Incidence		14.4		29.4		41.6		65.0		88.1		47.4
Liver	C22	n	7	(12.1)	4	(4.5)	7	(4.7)	18	(6.3)	21	(4.7)	57	(5.6)
		Incidence		16.8		14.7		24.2		53.2		59.7		34.2
Biliary Tract	C23-C24	n	1	(1.7)	1	(1.1)	4	(2.7)	6	(2.1)	14	(3.2)	26	(2.5)
		Incidence		2.4		3.7		13.9		17.7		39.8		15.6
Pancreas	C25	n	2	(3.4)	3	(3.4)	6	(4.1)	12	(4.2)	13	(2.9)	36	(3.5)
		Incidence		4.8		11.0		20.8		35.5		37.0		21.6
Larynx	C32	n	1	(1.7)	1	(1.1)	3	(2.0)	7	(2.4)	7	(1.6)	19	(1.9)
		Incidence		2.4		3.7		10.4		20.7		19.9		11.4
Lung	C33-C34	n	2	(3.4)	10	(11.4)	18	(12.2)	33	(11.5)	78	(17.6)	141	(13.8)
		Incidence		4.8		36.8		62.3		97.5		221.7		84.5
Prostate	C61	n	1	(1.7)	1	(1.1)	3	(2.0)	6	(2.1)	8	(1.8)	19	(1.9)
		Incidence		2.4		3.7		10.4		17.7		22.7		11.4
Kidney	C64-C66, C68	n	1	(1.7)	6	(6.8)	5	(3.4)	9	(3.1)	15	(3.4)	36	(3.5)
		Incidence		2.4		22.1		17.3		26.6		42.6		21.6
Bladder	C67	n	2	(3.4)	2	(2.3)	2	(1.4)	6	(2.1)	16	(3.6)	28	(2.7)
		Incidence		4.8		7.4		6.9		17.7		45.5		16.8
Thyroid	C73	n	0	(0.0)	1	(1.1)	2	(1.4)	2	(0.7)	0	(0.0)	5	(0.5)
		Incidence		0.0		3.7		6.9		5.9		0.0		3.0
Leukemia	- ^‡^	n	3	(5.2)	1	(1.1)	3	(2.0)	4	(1.4)	5	(1.1)	16	(1.6)
		Incidence		7.2		3.7		10.4		11.8		14.2		9.6

Women														
Person-years			39,096		29,020		33,783		39,786		41,281		182,965	

All sites	C00-C80	n	81	(100)	76	(100)	106	(100)	181	(100)	251	(100)	695	(100)
		Incidence		207.2		261.9		313.8		454.9		608.0		379.9
Esophagus	C15	n	0	(0.0)	0	(0.0)	4	(3.8)	0	(0.0)	3	(1.2)	7	(1.0)
		Incidence		0.0		0.0		11.8		0.0		7.3		3.8
Stomach	C16	n	14	(17.3)	14	(18.4)	19	(17.9)	29	(16.0)	47	(18.7)	123	(17.7)
		Incidence		35.8		48.2		56.2		72.9		113.9		67.2
Colon	C18	n	2	(2.5)	7	(9.2)	13	(12.3)	15	(8.3)	36	(14.3)	73	(10.5)

		Incidence		5.1		24.1		38.5		37.7		87.2		39.9
Rectum	C19-C21	n	5	(6.2)	5	(6.6)	11	(10.4)	14	(7.7)	14	(5.6)	49	(7.1)
		Incidence		12.8		17.2		32.6		35.2		33.9		26.8
Liver	C22	n	0	(0.0)	0	(0.0)	0	(0.0)	6	(3.3)	8	(3.2)	14	(2.0)
		Incidence		0.0		0.0		0.0		15.1		19.4		7.7
Biliary Tract	C23-C24	n	0	(0.0)	2	(2.6)	3	(2.8)	6	(3.3)	12	(4.8)	23	(3.3)
		Incidence		0.0		6.9		8.9		15.1		29.1		12.6
Pancreas	C25	n	0	(0.0)	2	(2.6)	0	(0.0)	10	(5.5)	12	(4.8)	24	(3.5)
		Incidence		0.0		6.9		0.0		25.1		29.1		13.1
Larynx	C32	n	0	(0.0)	0	(0.0)	1	(0.9)	0	(0.0)	0	(0.0)	1	(0.1)
		Incidence		0.0		0.0		3.0		0.0		0.0		0.5
Lung	C33-C34	n	2	(2.5)	6	(7.9)	7	(6.6)	17	(9.4)	22	(8.8)	54	(7.8)
		Incidence		5.1		20.7		20.7		42.7		53.3		29.5
Breast	C50	n	36	(44.4)	19	(25.0)	18	(17.0)	32	(17.7)	31	(12.4)	136	(19.6)
		Incidence		92.1		65.5		53.3		80.4		75.1		74.3
Uterus	C53-C55	n	4	(4.9)	5	(6.6)	7	(6.6)	5	(2.8)	6	(2.4)	27	(3.9)
		Incidence		10.2		17.2		20.7		12.6		14.5		14.8
Cervix Uteri	C53	n	3	(3.7)	2	(2.6)	4	(3.8)	1	(0.6)	3	(1.2)	13	(1.9)
		Incidence		7.7		6.9		11.8		2.5		7.3		7.1
Corpus Uteri	C54	n	1	(1.2)	2	(2.6)	3	(2.8)	4	(2.2)	2	(0.8)	12	(1.7)
		Incidence		2.6		6.9		8.9		10.1		4.8		6.6
Ovary	C56	n	5	(6.2)	1	(1.3)	6	(5.7)	6	(3.3)	4	(1.6)	22	(3.2)
		Incidence		12.8		3.4		17.8		15.1		9.7		12.0
Kidney	C64-C66, C68	n	0	(0.0)	0	(0.0)	0	(0.0)	6	(3.3)	9	(3.6)	15	(2.2)
		Incidence		0.0		0.0		0.0		15.1		21.8		8.2
Bladder	C67	n	1	(1.2)	0	(0.0)	0	(0.0)	7	(3.9)	6	(2.4)	14	(2.0)
		Incidence		2.6		0.0		0.0		17.6		14.5		7.7
Thyroid	C73	n	8	(9.9)	8	(10.5)	10	(9.4)	13	(7.2)	13	(5.2)	52	(7.5)
		Incidence		20.5		27.6		29.6		32.7		31.5		28.4
Leukemia	- ^‡^	n	1	(1.2)	1	(1.3)	1	(0.9)	1	(0.6)	1	(0.4)	5	(0.7)
		Incidence		2.6		3.4		3.0		2.5		2.4		2.7

† : per 100,000 person-years

‡ : M-9800 through M-9941

Percentages in parentheses

### The Association between Smoking and Lung Cancer

Of 22,836 male subjects who answered the questionnaire, we excluded 424 subjects with past history of cancer on the basis of cancer registry data only (281 subjects), self-report only (49 subjects) or both (94 subjects). We further excluded 717 subjects because of incomplete responses for smoking. Finally, we included 21,695 subjects with 129 cases of lung cancer incidence in this analysis.

The prevalence of current smokers, past smokers and never smokers among men were 61.8%, 19.8%, and 18.4%, respectively. Table 2 shows relative risks for the incidence of lung cancer according to smoking status. Age-adjusted relative risk of lung cancer increased significantly for current smokers compared with never smokers, and the risk remained after multivariable adjustment. The risk for past smokers was also increased but was lower than current smokers and not statistically significant. For current smokers, the risk of lung cancer had statistically significant dose-response relationship with number of cigarettes per day and pack-years. For those who smoked 40 cigarettes and over per day, the relative risk was 6.43 (95% confidence intervals [CI], 2.60-15.87) after multivariable adjustment. The risk of lung cancer had a tendency to increase with higher pack-years among current smokers. The multivariable adjusted relative risk for 60 pack-years and over was 4.27 (1.70-10.72), lower than those for 40-59 pack-years. However, 95% CIs overlapped each other.

**Table 2.** Lung cancer risk by smoking intensity in men.

		Person-years	No. of cases	RR1	(95% CI)	RR2	(95% CI)
Never smoker		29,464	8	1.00		1.00	
Past smoker		31,265	18	1.66	(0.72 - 3.81)	1.50	(0.65 - 3.48)
Current smoker		97,859	103	3.82	(1.86 - 7.85)	3.61	(1.75 - 7.46)

Number of cigarettes per day ^1^	0-19	23,597	17	2.10	(0.91 - 4.88)	1.98	(0.85 - 4.61)
	20-29	41,091	52	4.47	(2.12 - 9.40)	4.26	(2.01 - 9.02)
	30-39	15,709	17	5.12	(2.21 - 11.87)	4.98	(2.13 - 11.62)
	40+	11,168	12	6.62	(2.70 - 16.24)	6.43	(2.60 - 15.87)
	P for trend			< 0.001		< 0.001	

Pack-years ^1,2^	0-19	16,983	7	2.01	(0.73 - 5.55)	1.93	(0.70 - 5.33)
	20-39	46,986	41	3.59	(1.68 - 7.66)	3.40	(1.59 - 7.29)
	40-59	18,416	37	5.70	(2.65 - 12.27)	5.50	(2.54 - 11.90)
	60+	6,123	11	4.46	(1.79 - 11.10)	4.27	(1.70 - 10.72)
	P for trend			< 0.001		< 0.001	

RR1: adjusted for age in years

RR2: Adjusted for age in years; education (up to 15 years of age, 16-18, 19 years or older); alcohol drinking (never drinkers, past drinkers, or current drinkers who consumed less than 22.8g, 22.8-45.5g, or 45.6 g or more alcohol per day); walking time per day (less than 1 hour, or 1 hour or longer); consumption frequencies of green vegetables and oranges (almost daily, 3-4 times per week, 1-2 times per week, or 1-2 times per month or less often).

^1^ Current smokers only

^2^ (Number of cigarettes smoked per day × years of smoking)/20

## DISCUSSION

We identified 1,718 incident cancer cases (1,023 in men and 695 in women) by record linkage with regional cancer registries. As shown in Table 1, gastric cancer was the most common cancer among men in this cohort, followed by lung, and colon cancer. In women, breast cancer and gastric cancer were principal sites of cancer. In regard to these sites, it seems to be possible for us to investigate the effect of the health-related lifestyle on each site. The characteristics of cancer occurrence in this cohort were nearly consistent with a pattern of Miyagi prefecture in the same age group.^9^

As shown in Table 2, the risk of lung cancer was elevated in both past smokers and current smokers, and had dose-response relationship between the number of cigarettes per day, pack-years and lung cancer incidence. However, the risk of smokers in this cohort was lower than those of the subjects exposed to the same smoking intensity in the previous studies conducted in Western countries.^2^^,^^3^^,^^10^^,^^11^ Also, the relative risks of lung cancer among Japanese smokers observed in other prospective studies were lower than those presented in Western countries.^4^^,^^12^^,^^13^ Whether the difference in other smoking characteristics such as inhalation or genetic susceptibility is associated with a lower relative risk of lung cancer by smoking among the Japanese is unclear. Further studies are warranted.

By record linkage with regional cancer registry data, we are able to analyze the relationship between various life styles and cancer incidence in this cohort. Especially, the information of histological types and clinical stage in cancer registry data is useful for the detailed analysis to elucidate the effect of life-style factors on cancer occurrence.
